# 3-(6-Methyl-2-pyrid­yl)-2-phen­oxy-3,4-dihydro-1,3,2-benzoxaza­phosphirine 2-oxide

**DOI:** 10.1107/S1600536809029018

**Published:** 2009-07-25

**Authors:** Rajni Kant, Sabeta Kohli, Lovely Sarmal, M. Krishnaiah, V. H. H. Surendra Babu

**Affiliations:** a Department of Physics, University of Jammu, Jammu Tawi 180 006, India; b Department of Physics, S.V. University, Tirupati 517 502, India

## Abstract

In the title compound, C_19_H_17_N_2_O_3_P, the six-membered 1,3,2-oxaza­phospho­rine ring adopts a twist-boat conformation with the phosphoryl O atom in an equatorial position. The P=O(oxide) bond length is 1.457 (1) Å and the average value of the P—O distances is 1.588 Å. The crystal structure is stabilized by C—H⋯O and C—H⋯π inter­actions.

## Related literature

For the chemistry of organophospho­rus heterocyclic compounds, see: Przybylski *et al.* (1977 ▶); Riffel *et al.* (1984 ▶); Kleemann & Fluck (1985 ▶); Bettemann *et al.* (1987 ▶); He *et al.* (1998 ▶).
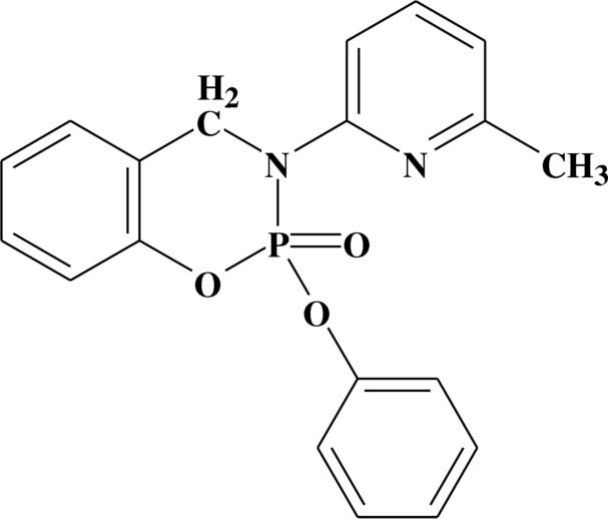


## Experimental

### 

#### Crystal data

C_19_H_17_N_2_O_3_P
*M* *_r_* = 352.33
Monoclinic, 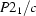

*a* = 9.2852 (7) Å
*b* = 14.2972 (11) Å
*c* = 13.3446 (8) Å
β = 104.545 (7)°
*V* = 1714.8 (2) Å^3^
*Z* = 4
Mo *K*α radiation
μ = 0.18 mm^−1^
*T* = 293 K
0.30 × 0.24 × 0.18 mm

#### Data collection

Oxford Diffraction Xcalibur diffractometer
Absorption correction: none
14565 measured reflections
4922 independent reflections
4064 reflections with *I* > 2σ(*I*)
*R*_int_ = 0.017

#### Refinement

*R*[*F* ^2^ > 2σ(*F* ^2^)] = 0.055
*wR*(*F* ^2^) = 0.149
*S* = 1.08
4922 reflections
227 parameters
H-atom parameters constrained
Δρ_max_ = 0.53 e Å^−3^
Δρ_min_ = −0.35 e Å^−3^

### 

Data collection: *CrysAlis Pro* (Oxford Diffraction, 2007 ▶); cell refinement: *CrysAlis Pro*; data reduction: *CrysAlis RED* (Oxford Diffraction, 2007 ▶); program(s) used to solve structure: *SHELXS86* (Sheldrick, 2008 ▶); program(s) used to refine structure: *SHELXL97* (Sheldrick, 2008 ▶); molecular graphics: *ORTEP-3 for Windows* (Farrugia, 1997 ▶); software used to prepare material for publication: *WinGX* (Farrugia, 1999 ▶).

## Supplementary Material

Crystal structure: contains datablocks global, I. DOI: 10.1107/S1600536809029018/jh2088sup1.cif

Structure factors: contains datablocks I. DOI: 10.1107/S1600536809029018/jh2088Isup2.hkl

Additional supplementary materials:  crystallographic information; 3D view; checkCIF report

## Figures and Tables

**Table 1:** Hydrogen-bond geometry (Å, °)

*D*—H⋯*A*	*D*—H	H⋯*A*	*D*⋯*A*	*D*—H⋯*A*
C4—H4⋯O3^i^	0.93	2.57	3.497 (3)	174
C9—H9⋯O2^i^	0.93	2.82	3.706 (2)	159
C7—H7*A*⋯O3^i^	0.97	2.85	3.425 (2)	119
C3—H3⋯O1^ii^	0.93	2.81	3.373 (3)	120
C11—H11⋯O3^iii^	0.93	2.66	3.561 (2)	165
C6—H6*C*⋯O2^iv^	0.96	2.81	3.443 (3)	124
C18—H18⋯N2^iv^	0.93	2.92	3.818 (5)	163
C10—H10⋯*Cg*2^iii^	0.93	2.78	3.437 (2)	128

Symmetry codes: (i) 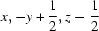; (ii) 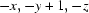; (iii) 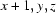; (iv) 
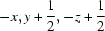
. *Cg*2 is the centroid of the N1,C1–C5 ring.

**Fig. 1.**
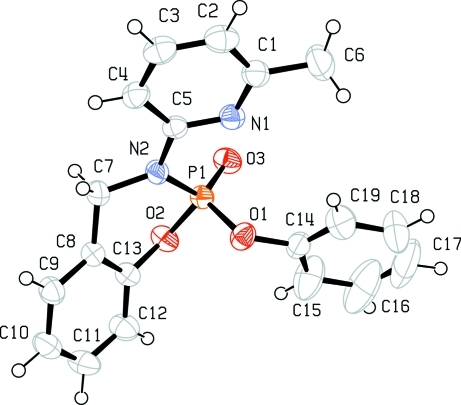
View of (I) (50% probability displacement ellipsoids)

**Fig. 2.**
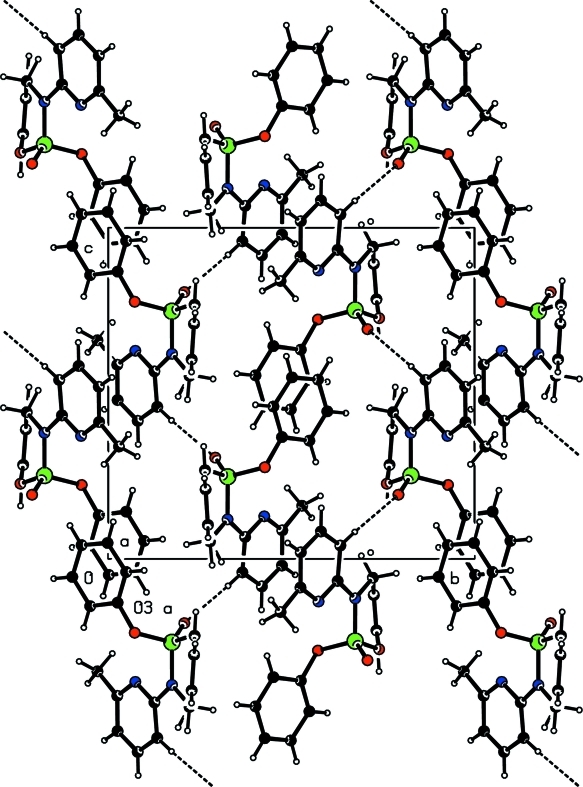
Depiction of C—H..O interactions in the title compound(I)

## supplementary crystallographic information

### Comment

Organophosphorus heterocycle compounds containing O and N in a six membered ring have gained much attention because of anti-cancer, anti-tumor and their most significant biological activities as pesticides and drugs(Riffel *et al.*1984, Kleemann *et al.*1985, Bettemann et *al. *1987, He *et al.*1998).).

Various compounds of this class *viz*. cyclophosphamide(2-bis(2-chloroethyl)amino tetrahydro-2*H*-1,3,2-oxazaphosphorine 2-oxide),ifosfamide(3-(2-chloroethyl)-2-(2-chloroethylamino)tetrahydro-2*H*-1,3,1-oxazaphosphorine2-oxide) and trofosfamide(3-(2-chloroethyl)-2-(bis(2-chloroethyl)amino)tetrahydro-2*H*-1,3,2oxazaphosphorine2-oxide) act as antitumor agents (Przybylski *et al.*,1977).

Because of these significant properties of organophosphorus heterocycle compounds a new compound of this class, *i.e.*, 3-(6-methyl-pyrindin-2-yl)-2-phenoxy-3, 4-dihydrobenzo[*e*][1,3,2]oxazaphosphirine 2-oxide[I] has been synthesized and its crystal structure is reported here.

In the title compound, [C_19 _H_17_ N_2 _O_3_ P], the six membered oxazaphosphorine ring adopts a twist boat conformation with phosphoryl oxygen atom at equatorial position. The PO(3) bond length is 1.457 (1)Å and the average value of P—O distance is 1.588 Å. The crystal structure is stabilized by C—H···O and C—H···Π interactions.

### Experimental

The title compound was synthesized by adding a solution of phenylphosphorodichloridate (0.002 mole) in 25 ml of dry tetrahydrofuran dropwise over a period of twenty minutes to a stirred solution of 2-{[(6-methyl-2-pyridyl)amino]methyl}phenol (0.002 mole) and triethylamine (0.004 mole) in 30 ml of dry tetrahydrofuran at 0°C. After completion of the addition, the temperature of the reaction mixture was slowly raised to room temperature and stirred for 30 min. Later the reaction mixture was heated to 45–50°C and maintained at that temperature for three hours with stirring. Completion of the reaction was monitored by TLC analysis. Triethylamine- hydrochloride was separated from the reaction mixture by filtration and the solvent was removed under reduced pressure. The crude product was purified by column chromatography on silica gel (100–200 mesh) as adsorbent and ethyl acetate:hexane as eluent to afford pure product. Suitable colourless transparent rectangular crystals were obtained from 2-propanol by slow evaporation technique.

### Refinement

All hydrogen atoms were fixed as a riding model over their respective heavier atoms and refined isotropically with distance restraints 0.93–0.97 Å

### Crystal data

**Table:**

C_19_H_17_N_2_O_3_P	*F*(000) = 736
*M**_r_* = 352.33	*D*_x_ = 1.365 Mg m^−^^3^
Monoclinic, *P*2_1_/*c*	Mo *K*α radiation, λ = 0.71073 Å
Hall symbol: -P 2ybc	Cell parameters from 4064 reflections
*a* = 9.2852 (7) Å	θ = 3.2–30.1°
*b* = 14.2972 (11) Å	µ = 0.18 mm^−^^1^
*c* = 13.3446 (8) Å	*T* = 293 K
β = 104.545 (7)°	Rectangular, colourless
*V* = 1714.8 (2) Å^3^	0.30 × 0.24 × 0.18 mm
*Z* = 4	

### Data collection

**Table:**

Oxford Diffraction Xcalibur diffractometer	*R*_int_ = 0.017
ω–2θ scans	θ_max_ = 30.1°, θ_min_ = 3.2°
14565 measured reflections	*h* = −13→13
4922 independent reflections	*k* = −19→20
4064 reflections with *I* > 2σ(*I*)	*l* = −18→18

### Refinement

**Table:**

Refinement on *F*^2^	0 restraints
Least-squares matrix: full	0 constraints
*R*[*F*^2^ > 2σ(*F*^2^)] = 0.055	H-atom parameters constrained
*wR*(*F*^2^) = 0.149	*w* = 1/[σ^2^(*F*_o_^2^) + (0.0646*P*)^2^ + 0.9398*P*] where *P* = (*F*_o_^2^ + 2*F*_c_^2^)/3
*S* = 1.08	(Δ/σ)_max_ < 0.001
4922 reflections	Δρ_max_ = 0.53 e Å^−^^3^
227 parameters	Δρ_min_ = −0.35 e Å^−^^3^

### Special details

**Table:**

Geometry. All e.s.d.'s (except the e.s.d. in the dihedral angle between two l.s. planes) are estimated using the full covariance matrix. The cell e.s.d.'s are taken into account individually in the estimation of e.s.d.'s in distances, angles and torsion angles; correlations between e.s.d.'s in cell parameters are only used when they are defined by crystal symmetry. An approximate (isotropic) treatment of cell e.s.d.'s is used for estimating e.s.d.'s involving l.s. planes.

### Fractional atomic coordinates and isotropic or equivalent isotropic displacement parameters (Å^2^)

**Table:**

	*x*	*y*	*z*	*U*_iso_*/*U*_eq_	
O3	0.05137 (15)	0.29221 (11)	0.30611 (10)	0.0431 (3)	
P1	0.15450 (5)	0.32614 (3)	0.24817 (3)	0.03212 (13)	
O2	0.29805 (14)	0.26052 (10)	0.27286 (10)	0.0408 (3)	
N2	0.10589 (16)	0.32584 (11)	0.11909 (11)	0.0342 (3)	
N1	−0.08432 (16)	0.42593 (11)	0.12856 (11)	0.0366 (3)	
O1	0.22025 (15)	0.42756 (10)	0.27690 (10)	0.0409 (3)	
C4	−0.0855 (2)	0.36620 (14)	−0.04067 (13)	0.0391 (4)	
H4	−0.0437	0.3271	−0.0814	0.047*	
C13	0.40587 (19)	0.26785 (13)	0.21602 (14)	0.0358 (4)	
C5	−0.02466 (18)	0.37381 (12)	0.06657 (13)	0.0320 (3)	
C8	0.35786 (19)	0.27514 (13)	0.10910 (14)	0.0363 (4)	
C3	−0.2102 (2)	0.41944 (15)	−0.08287 (15)	0.0450 (4)	
H3	−0.2522	0.4182	−0.1539	0.054*	
C2	−0.2726 (2)	0.47435 (15)	−0.02010 (17)	0.0471 (5)	
H2	−0.3565	0.5103	−0.0482	0.057*	
C9	0.4650 (2)	0.27788 (16)	0.05260 (15)	0.0439 (4)	
H9	0.436	0.2836	−0.0191	0.053*	
C1	−0.2078 (2)	0.47521 (14)	0.08637 (16)	0.0414 (4)	
C11	0.6595 (2)	0.26582 (18)	0.21010 (19)	0.0546 (5)	
H11	0.7603	0.2627	0.2432	0.065*	
C14	0.2129 (2)	0.47435 (14)	0.36794 (15)	0.0417 (4)	
C7	0.1947 (2)	0.27754 (18)	0.05690 (16)	0.0496 (5)	
H7A	0.1803	0.309	−0.0093	0.06*	
H7B	0.1586	0.2139	0.0436	0.06*	
C10	0.6146 (2)	0.27217 (16)	0.10275 (18)	0.0482 (5)	
H10	0.6856	0.2726	0.0644	0.058*	
C12	0.5546 (2)	0.26421 (17)	0.26780 (16)	0.0499 (5)	
H12	0.5836	0.2608	0.3397	0.06*	
C19	0.1181 (3)	0.5491 (2)	0.3572 (3)	0.0860 (10)	
H19	0.0597	0.5665	0.2926	0.103*	
C6	−0.2732 (3)	0.5295 (2)	0.1604 (2)	0.0663 (7)	
H6A	−0.2875	0.4887	0.2143	0.099*	
H6B	−0.3673	0.5554	0.1241	0.099*	
H6C	−0.2068	0.5792	0.1904	0.099*	
C16	0.2972 (7)	0.5017 (3)	0.5483 (3)	0.1263 (18)	
H16	0.3597	0.4863	0.6122	0.152*	
C18	0.1120 (5)	0.5980 (3)	0.4456 (5)	0.136 (2)	
H18	0.0477	0.6486	0.4405	0.163*	
C17	0.2005 (7)	0.5726 (4)	0.5416 (4)	0.131 (2)	
H17	0.1929	0.6044	0.6008	0.157*	
C15	0.3051 (4)	0.4509 (2)	0.4605 (2)	0.0841 (10)	
H15	0.3724	0.4019	0.4653	0.101*	

### Atomic displacement parameters (Å^2^)

**Table:**

	*U*^11^	*U*^22^	*U*^33^	*U*^12^	*U*^13^	*U*^23^
O3	0.0409 (7)	0.0518 (8)	0.0405 (7)	−0.0031 (6)	0.0173 (5)	0.0077 (6)
P1	0.0333 (2)	0.0364 (2)	0.0278 (2)	−0.00139 (17)	0.00979 (15)	0.00310 (16)
O2	0.0383 (6)	0.0486 (8)	0.0376 (6)	0.0065 (6)	0.0135 (5)	0.0127 (6)
N2	0.0328 (7)	0.0418 (8)	0.0282 (6)	0.0032 (6)	0.0079 (5)	−0.0012 (6)
N1	0.0345 (7)	0.0399 (8)	0.0352 (7)	0.0003 (6)	0.0083 (6)	−0.0003 (6)
O1	0.0492 (7)	0.0427 (7)	0.0329 (6)	−0.0091 (6)	0.0145 (5)	−0.0034 (5)
C4	0.0400 (9)	0.0458 (10)	0.0310 (8)	−0.0047 (8)	0.0080 (7)	0.0007 (7)
C13	0.0346 (8)	0.0349 (8)	0.0392 (8)	0.0019 (7)	0.0117 (7)	0.0049 (7)
C5	0.0308 (7)	0.0337 (8)	0.0314 (7)	−0.0042 (6)	0.0078 (6)	0.0019 (6)
C8	0.0342 (8)	0.0379 (9)	0.0377 (8)	0.0003 (7)	0.0107 (7)	−0.0025 (7)
C3	0.0459 (10)	0.0507 (11)	0.0339 (9)	−0.0058 (8)	0.0018 (7)	0.0082 (8)
C2	0.0395 (10)	0.0432 (10)	0.0534 (11)	0.0020 (8)	0.0017 (8)	0.0109 (9)
C9	0.0438 (10)	0.0511 (11)	0.0397 (9)	0.0029 (8)	0.0160 (8)	−0.0003 (8)
C1	0.0385 (9)	0.0375 (9)	0.0475 (10)	0.0004 (7)	0.0096 (7)	0.0000 (8)
C11	0.0326 (9)	0.0686 (15)	0.0609 (13)	0.0015 (9)	0.0089 (9)	0.0047 (11)
C14	0.0473 (10)	0.0404 (10)	0.0404 (9)	−0.0088 (8)	0.0165 (8)	−0.0073 (8)
C7	0.0351 (9)	0.0737 (15)	0.0393 (9)	0.0042 (9)	0.0081 (7)	−0.0191 (10)
C10	0.0389 (9)	0.0512 (12)	0.0597 (12)	−0.0003 (8)	0.0221 (9)	0.0022 (10)
C12	0.0397 (10)	0.0657 (14)	0.0419 (10)	0.0061 (9)	0.0055 (8)	0.0082 (9)
C19	0.0632 (16)	0.077 (2)	0.114 (3)	0.0136 (15)	0.0152 (16)	−0.0319 (19)
C6	0.0618 (14)	0.0702 (17)	0.0664 (15)	0.0237 (13)	0.0150 (12)	−0.0107 (13)
C16	0.220 (5)	0.113 (3)	0.0428 (15)	−0.033 (4)	0.028 (2)	−0.0242 (19)
C18	0.104 (3)	0.113 (3)	0.209 (6)	−0.006 (3)	0.075 (4)	−0.095 (4)
C17	0.190 (5)	0.115 (3)	0.124 (4)	−0.075 (4)	0.104 (4)	−0.083 (3)
C15	0.129 (3)	0.0735 (18)	0.0397 (12)	0.0134 (18)	0.0022 (14)	−0.0051 (12)

### Geometric parameters (Å, °)

**Table:**

O3—P1	1.4567 (13)	C1—C6	1.500 (3)
P1—O1	1.5825 (14)	C11—C12	1.385 (3)
P1—O2	1.5953 (14)	C11—C10	1.391 (3)
P1—N2	1.6675 (14)	C11—H11	0.93
O2—C13	1.404 (2)	C14—C15	1.356 (3)
N2—C5	1.416 (2)	C14—C19	1.369 (4)
N2—C7	1.479 (2)	C7—H7A	0.97
N1—C5	1.333 (2)	C7—H7B	0.97
N1—C1	1.343 (2)	C10—H10	0.93
O1—C14	1.404 (2)	C12—H12	0.93
C4—C3	1.382 (3)	C19—C18	1.385 (5)
C4—C5	1.405 (2)	C19—H19	0.93
C4—H4	0.93	C6—H6A	0.96
C13—C12	1.381 (3)	C6—H6B	0.96
C13—C8	1.388 (2)	C6—H6C	0.96
C8—C9	1.391 (2)	C16—C17	1.342 (7)
C8—C7	1.501 (3)	C16—C15	1.396 (5)
C3—C2	1.377 (3)	C16—H16	0.93
C3—H3	0.93	C18—C17	1.384 (8)
C2—C1	1.397 (3)	C18—H18	0.93
C2—H2	0.93	C17—H17	0.93
C9—C10	1.385 (3)	C15—H15	0.93
C9—H9	0.93		
			
O3—P1—O1	116.20 (8)	C10—C11—H11	119.9
O3—P1—O2	108.72 (8)	C15—C14—C19	122.0 (3)
O1—P1—O2	103.73 (8)	C15—C14—O1	120.9 (2)
O3—P1—N2	120.26 (8)	C19—C14—O1	116.8 (2)
O1—P1—N2	103.92 (7)	N2—C7—C8	112.96 (15)
O2—P1—N2	101.96 (7)	N2—C7—H7A	109
C13—O2—P1	121.11 (11)	C8—C7—H7A	109
C5—N2—C7	118.48 (14)	N2—C7—H7B	109
C5—N2—P1	119.09 (12)	C8—C7—H7B	109
C7—N2—P1	122.43 (12)	H7A—C7—H7B	107.8
C5—N1—C1	118.50 (15)	C9—C10—C11	120.42 (18)
C14—O1—P1	123.14 (12)	C9—C10—H10	119.8
C3—C4—C5	117.00 (18)	C11—C10—H10	119.8
C3—C4—H4	121.5	C13—C12—C11	118.40 (19)
C5—C4—H4	121.5	C13—C12—H12	120.8
C12—C13—C8	122.67 (17)	C11—C12—H12	120.8
C12—C13—O2	119.08 (16)	C14—C19—C18	117.9 (4)
C8—C13—O2	118.21 (15)	C14—C19—H19	121
N1—C5—C4	123.51 (16)	C18—C19—H19	121
N1—C5—N2	113.65 (14)	C1—C6—H6A	109.5
C4—C5—N2	122.83 (16)	C1—C6—H6B	109.5
C13—C8—C9	118.07 (16)	H6A—C6—H6B	109.5
C13—C8—C7	120.34 (16)	C1—C6—H6C	109.5
C9—C8—C7	121.58 (17)	H6A—C6—H6C	109.5
C2—C3—C4	120.22 (18)	H6B—C6—H6C	109.5
C2—C3—H3	119.9	C17—C16—C15	120.8 (4)
C4—C3—H3	119.9	C17—C16—H16	119.6
C3—C2—C1	118.98 (18)	C15—C16—H16	119.6
C3—C2—H2	120.5	C17—C18—C19	120.9 (4)
C1—C2—H2	120.5	C17—C18—H18	119.5
C10—C9—C8	120.23 (18)	C19—C18—H18	119.5
C10—C9—H9	119.9	C16—C17—C18	119.4 (3)
C8—C9—H9	119.9	C16—C17—H17	120.3
N1—C1—C2	121.71 (18)	C18—C17—H17	120.3
N1—C1—C6	116.08 (18)	C14—C15—C16	118.8 (4)
C2—C1—C6	122.20 (19)	C14—C15—H15	120.6
C12—C11—C10	120.19 (18)	C16—C15—H15	120.6
C12—C11—H11	119.9		

### Hydrogen-bond geometry (Å, °)

**Table:**

*D*—H···*A*	*D*—H	H···*A*	*D*···*A*	*D*—H···*A*
C4—H4···O3^i^	0.93	2.57	3.497 (3)	174
C9—H9···O2^i^	0.93	2.82	3.706 (2)	159
C7—H7A···O3^i^	0.97	2.85	3.425 (2)	119
C3—H3···O1^ii^	0.93	2.81	3.373 (3)	120
C11—H11···O3^iii^	0.93	2.66	3.561 (2)	165
C6—H6C···O2^iv^	0.96	2.81	3.443 (3)	124
C18—H18···N2^iv^	0.93	2.92	3.818 (5)	163
C10—H10···Cg2^iii^	0.93	2.78	3.437 (2)	128

Symmetry codes: (i) *x*, −*y*+1/2, *z*−1/2; (ii) −*x*, −*y*+1, −*z*; (iii) *x*+1, *y*, *z*; (iv) −*x*, *y*+1/2, −*z*+1/2.
